# A Novel Algorithm for Thickness Prediction in Incremental Sheet Metal Forming

**DOI:** 10.3390/ma15031201

**Published:** 2022-02-04

**Authors:** Yuhuai Wang, Lidong Wang, Huixi Zhang, Yong Gu, Yaokun Ye

**Affiliations:** 1 Qianjiang College, Hangzhou Normal University, Hangzhou 310018, China; yhwang@hznu.edu.cn (Y.W.); wld@huqc.edu.cn (L.W.); 20050021@hznu.edu.cn (H.Z.); 2 Sinofork Equipment Co., Ltd., Huzhou 313300, China; yeyaokun_zl@163.com

**Keywords:** incremental sheet metal forming, thickness prediction, model, NURBS

## Abstract

Incremental sheet metal forming characterized as increased flexibility and local plastic deformation is well suitable for low-production-run manufacturing and a new sample trial production of complex shapes. Thickness thinning is still an obstacle to the application of incremental forming. In this study, a novel mathematical algorithm based on a non-uniform rational B-spline (NURBS) surface was proposed and implemented which focuses on predicting and calculating the final thickness for arbitrary parts in incremental forming. In order to evaluate the validity of the proposed model, the finite element simulation and forming experiments of three kinds of parts, such as truncated cones, truncated pyramids and ellipsoid parts, were conducted. The thickness of theoretical prediction was compared with that of finite element simulation and experiment, and good agreements were obtained. The results show that the proposed model and the method are effective and robust for predicting the thickness of the formed parts in incremental sheet metal forming.

## 1. Introduction

Incremental sheet metal forming (ISMF) is an advanced rapid die-less flexible sheet forming technology for prototyping and low-production-run manufacturing of almost arbitrary 3D shapes. A variety of incremental forming (IF) technologies have been evolved, such as single-point incremental forming (SPIF), two-point incremental forming (TPIF) [1], double-sided incremental forming (DSIF) [2] and three opposite point incremental forming (TOPIF) [3]. The remarkable feature of the ISMF is that the sheet will be continuously thinned in the forming area [4]. During the ISMF process, thickness thinning will be induced as the localized plastic deformation is accumulated to produce the final part. So, thickness distribution is a critical factor for designing and controlling the incremental forming operations [5,6]. It is known that the sine law, verified on truncated cones, truncated pyramids and other simple formed parts is a concise model for prediction of thickness in the IF process [7,8,9,10,11,12]. Bambach presented a variant of the sine law by assuming that the sheet deforms along the normal direction of the smooth transient surface [13]. Malhotra et al. introduced a squeezing coefficient into the sine law for thickness prediction in DSIF [14]. Salem et al. presented an algorithm to predict and calculate the actual thickness by using the plane normal vector of the distorted element across the sheet [15]. Choi and Lee presented a mathematical prediction model of thickness distribution of the hybrid forming of ISMF and stretch forming based on the conventional sine law [16]. However, these methods have a great disadvantage in that they have a large number of calculations for the thickness prediction of parts with varied forming angles. Furthermore, they are not available for the thickness prediction of complicated parts with random shapes which are not represented directly by the mathematical function, such as F(x,y,z)=0.

The present study focused on developing a general model based on NURBS surface, which can be used to predict and calculate the final thickness for arbitrary parts. The freeform surface of an arbitrary part was reconstructed based on a non-uniform rational B-spline (NURBS) parametric surface. The first-order partial derivatives of the freeform surface were evaluated to solve the area. Based on the obtained area element, the thickness was calculated according to the principle of volume constancy in plastic deformation. In fact, the proposed model is an extension of the sine law. Taking the truncated cones with different forming angles, pyramid part and hemi-ellipsoid part for examples, experiments are performed to show that the proposed model can predict the final thickness for complicated parts with a random shape.

## 2. Mathematical Model and Methods for Thickness Calculation

In this work, the freeform surface of the forming part is described by using a NURBS surface which is a parametric polynomial surface.

A NURBS surface with the degrees of p and q along the parametric direction of u and v, respectively, can be defined as follows:

(1) $$P(x,y,z)=f(u,v)=\frac{\sum_{i=0}^{m}\sum_{j=0}^{n}N_{i,p}(u)N_{j,q}(v)\omega_{i,j}P_{i,j}}{\sum_{i=0}^{m}\sum_{j=0}^{n}N_{i,p}(u)N_{j,q}(v)\omega_{i,j}}\begin{array}{cc} & 0\leq u,v\leq 1 \end{array}$$

where P(x,y,z) is the arbitrary point on the NURBS surface. i=0,⋯,m, j=0,⋯,n, $P_{i,j}$ are the control points, $\omega_{i,j}$ are the weight coefficients, (m+1) and (n+1) are the amount of control points, and $N_{i,p}(u)$ and $N_{j,q}(v)$ are the B-spline basis functions of order p and q, respectively, defined over the knot vectors $U=\left[\underset{p+1}{\underset{︸}{0,\cdots ,0}},u_{p+1},\cdots ,u_{m},\underset{p+1}{\underset{︸}{1,\cdots ,1}}\right]$ and $V=\left[\underset{q+1}{\underset{︸}{0,\cdots ,0}},v_{q+1},\cdots ,v_{n},\underset{q+1}{\underset{︸}{1,\cdots ,1}}\right]$, which are calculated by:

$$N_{i,0}(u)=\{\begin{array}{cc} 1, & u_{i}\leq u\leq u_{i+1}\\ 0, & \mathrm{otherwise} \end{array}$$

$$N_{i,p}(u)=\frac{\left(u-u_{i})N_{i,p-1}(u\right)}{\left(u_{i+p}-u_{i}\right)}+\frac{\left(u_{i+p+1}-u)N_{i+1,p-1}(u\right)}{\left(u_{i+p+1}-u_{i+1}\right)}$$

$$\frac{0}{0}=0$$

According to the first fundamental form of a surface, the surface area can be expressed as:

(2) $$S(f)=\iint_{D}\left|f_{u}\times f_{v}\right|dudv\begin{array}{cc} & \left(u,v\right) \end{array}\in D$$

where D is the parameter domain, and $f_{u}$ and $f_{v}$ are the partial derivatives of the surface f with respect to the parametric variable u and v, respectively. $f_{u}\times f_{v}$ is just the cross product, which can be calculated as follows:

(3) $$f_{u}\times f_{v}=\frac{\partial (y,z)}{\partial (u,v)}\vec{x}+\frac{\partial (z,x)}{\partial (u,v)}\vec{y}+\frac{\partial (x,y)}{\partial (u,v)}\vec{z}$$

where $\frac{\partial (x,y)}{\partial (u,v)}=\left(\begin{array}{cc} \frac{\partial x}{\partial u} & \frac{\partial y}{\partial u}\\ \frac{\partial x}{\partial v} & \frac{\partial y}{\partial v} \end{array}\right)$, $\frac{\partial (y,z)}{\partial (u,v)}=\left(\begin{array}{cc} \frac{\partial y}{\partial u} & \frac{\partial z}{\partial u}\\ \frac{\partial y}{\partial v} & \frac{\partial z}{\partial v} \end{array}\right)$ and $\frac{\partial (z,x)}{\partial (u,v)}=\left(\begin{array}{cc} \frac{\partial z}{\partial u} & \frac{\partial x}{\partial u}\\ \frac{\partial z}{\partial v} & \frac{\partial x}{\partial v} \end{array}\right)$.

In differential notation, the surface area of the patch of the surface is:

(4) $$dS=\frac{\left|f_{u}\times f_{v}\right|}{\left|\frac{\partial (x,y)}{\partial (u,v)}\right|}dxdy$$

Let

$$W(u,v)=\sum_{i=0}^{n}\sum_{j=0}^{m}N_{i,p}(u)N_{j,q}(v)\omega_{i,j}$$

and

$$A(u,v)=\sum_{i=0}^{n}\sum_{j=0}^{m}N_{i,p}(u)N_{j,q}(v)\omega_{i,j}P_{i,j}$$

Then, we can obtain

$$\begin{array}{l} f_{u}\left(u,v\right)\\ =\frac{W\left(u,v\right)\sum_{i=0}^{n}\sum_{j=0}^{m}N_{i,p}^{\prime }\left(u\right)N_{j,q}\left(v\right)\omega_{i,j}P_{i,j}-A\left(u,v\right)\sum_{i=0}^{n}\sum_{j=0}^{m}N_{i,p}^{\prime }\left(u\right)N_{j,q}\left(v\right)\omega_{i,j}}{{\left[W\left(u,v\right)\right]}^{2}} \end{array}$$

$$\begin{array}{l} f_{v}\left(u,v\right)\\ =\frac{W\left(u,v\right)\sum_{i=0}^{n}\sum_{j=0}^{m}N_{i,p}\left(u\right)N_{j,q}^{\prime }\left(v\right)\omega_{i,j}P_{i,j}-A\left(u,v\right)\sum_{i=0}^{n}\sum_{j=0}^{m}N_{i,p}\left(u\right)N_{j,q}^{\prime }\left(v\right)\omega_{i,j}}{{\left[W\left(u,v\right)\right]}^{2}} \end{array}$$

where

$$N_{i,p}^{\prime }\left(u\right)=p\left[\frac{N_{i,p-1}\left(u\right)}{u_{i+p}-u_{i}}-\frac{N_{i+1,p-1}\left(u\right)}{u_{i+p+1}-u_{i+1}}\right]$$

$$N_{j,q}^{\prime }\left(v\right)=q\left[\frac{N_{j,q-1}\left(v\right)}{v_{j+q}-v_{j}}-\frac{N_{j+1,q-1}\left(v\right)}{v_{j+q+1}-v_{j+1}}\right]$$

Due to volume constancy in plastic deformation, we obtain

(5) $$t_{0}dxdy=tdS$$

where $t_{0}$ and t represent the thickness of the original blank sheet and the actual formed sheet, respectively.

Substitution of Equation (4) into Equation (5) gives:

(6) $$t=t_{0}\left|\frac{\partial (x,y)}{\partial (u,v)}\right|/\left|f_{u}\times f_{v}\right|$$

The algorithm presented in this study is rather general in predicting thickness. It holds for the thickness prediction of simple parts and complicated parts with a random shape. In fact, it can also be found that the proposed algorithm is the same as the sine law for the thickness prediction of parts with constant forming angle, such as conical shape and pyramid shape. In other words, its advantage is to predict and calculate the thickness of parts with a variable forming angle. In a few words, this algorithm is more convenient than the sine law to calculate the thickness once the surface equation of the desired component is obtained.

The procedure of the proposed mathematical modeling approach can be summarized as follows:

1. Get the Cartesian or coordinates of the discrete points;
2. Set the knot vectors;
3. Construct the NURBS surface;
4. Compute the first derivatives of the NURBS surface;
5. Compute the cross product of two partial derivatives;
6. Compute the final thickness.

## 3. Experimental Results and Discussion

**Figure 1:**
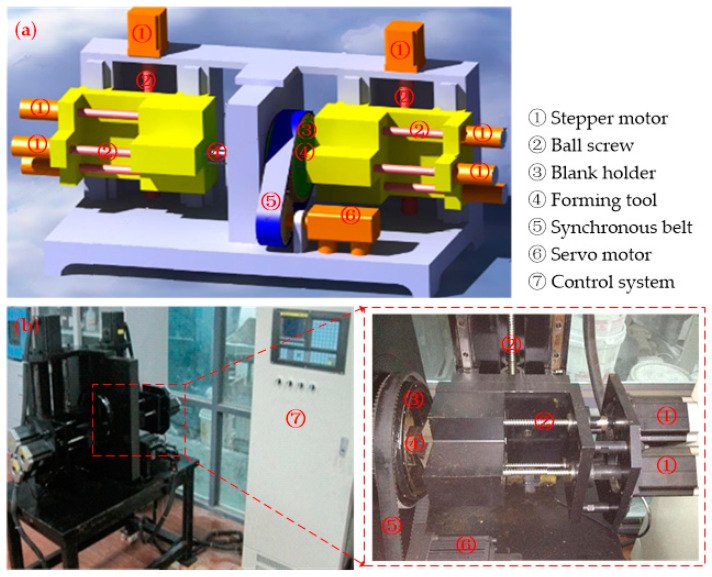
Incremental forming machine: (**a**) schematic of the mechanical structure; (**b**) fabricated forming machine.

Based on the above results, to verify the validity of the relation developed in Equation (6), the truncated cone part, the truncated pyramid part and the ellipsoid part have been designed and formed by a custom-designed and fabricated machine shown in Figure 1. Figure 1a shows the schematic of the mechanical structure. During the forming process, the blank holder driven by the servo motor and the sheet clamped on the blank holder rotate together. The forming tools driven by the stepper motors can move along the horizontal and vertical direction. The prototype of the deformation machine shown as Figure 1b was manufactured by Zhejiang University of Technology. This machine can be used to form the hyperbolic parts online by selecting and controlling different forming tools, benefiting from the design of its bilateral symmetrical structure. The maximum displacements of the forming head are 200 mm and 100 mm along the vertical and horizontal direction, respectively. The diameter of the annular blank holder is 160 mm, which determines the size of the blank sheet in the experiment. So, the diameter of the circular aluminum sheet used in experiments is determined to be 160 mm. The initial thickness of the aluminum sheet is 1 mm. The diameter of the forming tool with hemispherical head which is made of tool steel is 6 mm. In order to enable the final thickness to be as uniform as possible and obtain better surface quality, the oil lubrication was used during forming process in the following experiments. The material parameters of aluminum sheet and forming tool are shown in Table 1.

In order to better grasp the thickness distribution, the finite element simulation was performed using ANSYS/LS-DYNA. As the diameter of the aluminum sheet is 160 times than its thickness, the sheet can be regarded as a thin sheet in the simulation. The element type of the SHELL 163 was adopted for the aluminum sheet. Considering the influence of time and accuracy, the Belytschko–Wong–Chiang was used as the element formulation. Accordingly, the element type of SOLID 164 was adopted for the forming tool modeled as a rigid body. The Barlat model with the exponential anisotropic hardening assumption is defined as the constitutive model of aluminum sheet. The yield rule can be expressed as:

(7) $${\left(\sigma_{s}\right)}^{k}=(\alpha |C_{1}+C_{2}{|}^{k}+\alpha |C_{1}-C_{2}{|}^{k}+\beta |2C_{2}{|}^{k})/2$$

where $\sigma_{s}=\delta \varepsilon^{\eta }$ is the yield stress, δ and η are the constants determined by experiment, ε is the strain. k is the Barrat constant, $C_{1}=(\sigma_{1}-\lambda \sigma_{2})/2$, $C_{2}=\sqrt{C_{1}^{2}+\mu^{2}\tau_{12}^{2}}$, α, β, λ and μ are the constants of the anisotropic material. $\sigma_{1}$ and $\sigma_{2}$ are the principal stress, $\tau_{12}$ is the shear stress. Considering the use of oil lubrication in the experiment, the surface to surface contact in which the boundary friction with friction coefficient of 0.1 was introduced was defined between the aluminum sheet and the forming head. The outer edge of the aluminum sheet is constrained. The mesh size of the sheet metal deformation area and the forming tool are 1 mm and 2 mm, respectively.

In the experiments and simulations, some parameters including the coordinates of the starting point, radial feed rate, axial step down and rotational speed of the blank holder with sheet need to be set and input, according to the contour path. The forming parameters of the formed parts in this study are tabulated in Table 2. It should be noted that the coordination between sheet rotation and the feed of forming head needs to be planned and designed for pyramid and ellipsoid parts.

### 3.1. Case 1: Truncated Cone Shape

**Figure 2:**
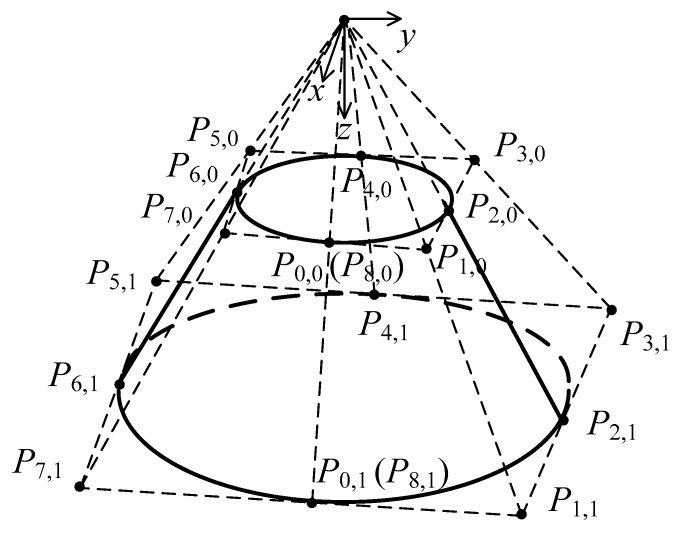
A truncated cone surface of degree 2 in each of its 2 parameters.

A cone as a 2 × 2 degree NURBS surface with 9 × 3 control points shown in Figure 2 can be constructed as:

(8) $$P(x,y,z)=f(u,v)=\frac{\sum_{i=0}^{8}\sum_{j=0}^{2}N_{i,2}(u)N_{j,2}(v)\omega_{i,j}P_{i,j}}{\sum_{i=0}^{8}\sum_{j=0}^{2}N_{i,2}(u)N_{j,2}(v)\omega_{i,j}}\begin{array}{cc} & 0\leq u,v\leq 1 \end{array}$$

where the knot vectors are $U_{1\times 12}=\left[0,0,0,0.25,0.25,0.5,0.5,0.75,0.75,1,1,1\right]$ and $V_{1\times 6}=\left[0,0,0,1,1,1\right]$. The array of weights is $\left[\omega_{i,j}\right]=\left[\begin{array}{c} 1,\sqrt{2}/2,1,\sqrt{2}/2,1,\sqrt{2}/2,1,\sqrt{2}/2,1\\ 1,\sqrt{2}/2,1,\sqrt{2}/2,1,\sqrt{2}/2,1,\sqrt{2}/2,1\\ 1,\sqrt{2}/2,1,\sqrt{2}/2,1,\sqrt{2}/2,1,\sqrt{2}/2,1 \end{array}\right]$.

**Figure 3:**
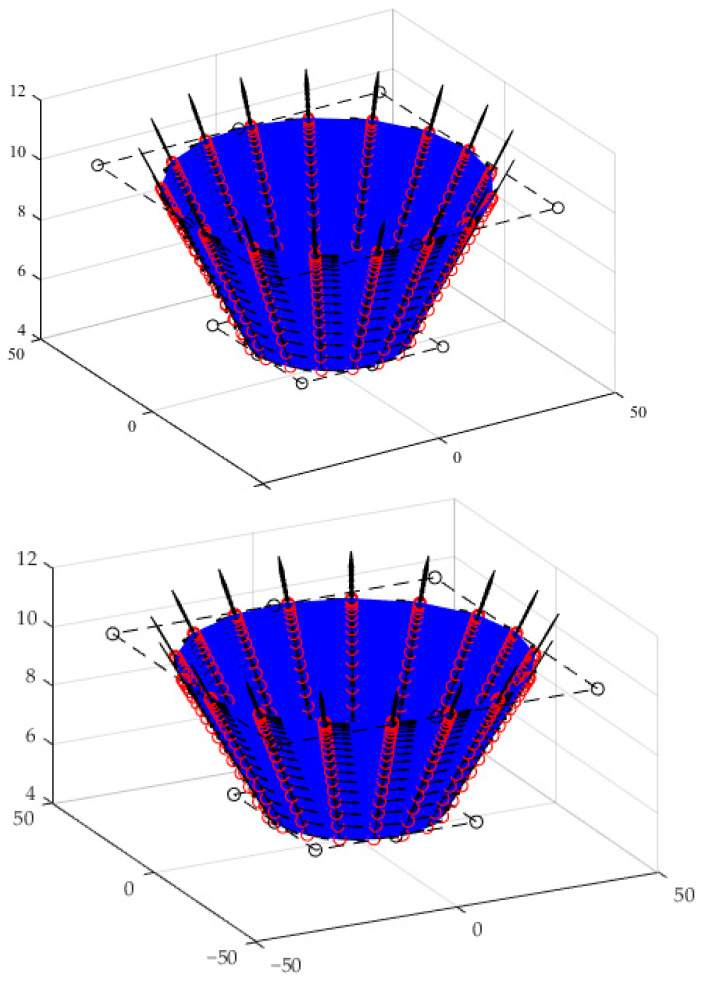
The first derivatives over the designed conical surface with forming angle of 76° and height of 5 mm.

Two kinds of truncated cones with forming angles of 76° and 53.2°, and corresponding heights of 5 mm and 15 mm, were designed by $[P_{0,0},P_{0,1},P_{0,2}]=\left[\begin{array}{ccc} 20 & 40 & 40\\ 0 & 0 & 0\\ 5 & 10 & 10 \end{array}\right]$ and $[P_{0,0},P_{0,1},P_{0,2}]=\left[\begin{array}{ccc} 10 & 30 & 30\\ 0 & 0 & 0\\ 7.5 & 22.5 & 22.5 \end{array}\right]$, respectively. The first derivatives over the designed conical surface with forming angle of 76° and height of 5 mm are shown in Figure 3.

**Figure 4:**
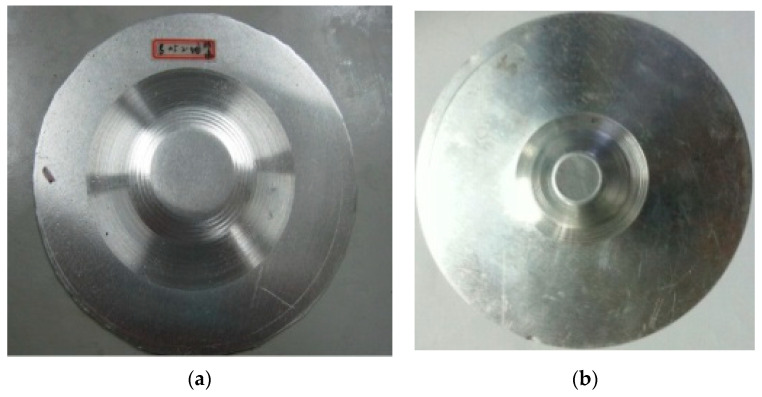
Formed truncated cones: (**a**) 76°; (**b**) 53.2°.

The two truncated cones with the different forming angles were formed by using the aluminum blank sheet with thickness of *t*_0_ = 1 mm. The step down of Δ*z* = 0.5 mm and Δ*z* = 0.75 mm shown in Table 2 were used in the tests, respectively. During the forming process, the oil lubricant was painted on the sheet to improve the surface quality by reducing the friction caused by the forming tool. The formed cones are shown in Figure 4.

**Figure 5:**
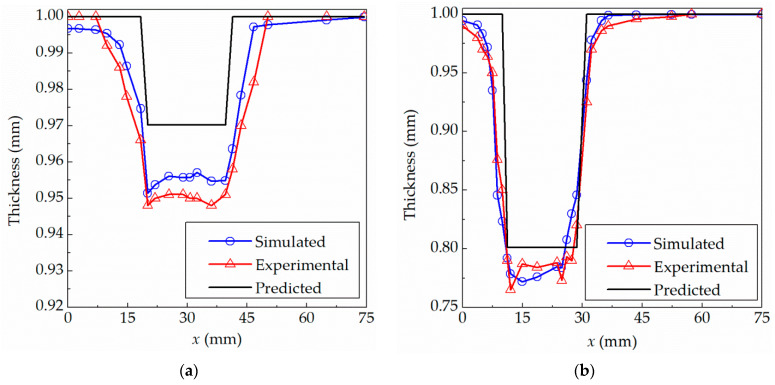
Comparison among experimental, simulated and theoretical predicted thickness for truncated cones: (**a**) 76°; (**b**) 53.2°.

In order to evaluate the proposed theoretical model, the comparison of theoretical, simulated and experimental thickness was performed. A micrometer with the accuracy of 0.01 mm was used to measure the thickness of the experimental sheet. In order to reduce the errors as much as possible, the formed parts are cut using wire cutting to obtain the measured sample, and then the average value of five measurements is taken as the actual thickness. The simulated thickness is extracted from the thickness fields based on LS-PrePost, which is solved using the finite element method (FEM). The theoretical thickness is calculated using the presented algorithm according to Equation (6). The corresponding thicknesses for the truncated cones are plotted in Figure 5 as a comparison.

Figure 5a shows the thickness profile of the truncated cone with a forming angle of 76° away from the central axis at *x* = 0 mm along the radial direction. The bottom surface of the truncated cone located within the range of *x* = 0–20 mm, which belongs to the unformed area, gradually moves downward as a whole, when the forming is carried out. The inclined wall located within the range of *x* = 20–40 mm is the formed area. The area over *x* = 40 mm, of which the outer edge is clamped by blank holder, belongs to the unformed area. The thickness of the formed region is basically unchanged because of the constant forming angle. The average thickness of experiment and simulation are 0.950 mm and 0.955 mm, respectively. The result predicted by this presented algorithm is 0.97 mm for the truncated cone with the forming angle of 76°, which is the same as the result of sin(76°) = 0.97 mm calculated using the traditional sine law. The thickness thinning was found in the transition regions between the unformed and formed areas. The center and outer edge of the sheet basically maintain the original thickness. The same situation was found in Figure 5b. The average thicknesses of the sheet in the formed area located within the range of *x* = 10–30 mm are 0.788 mm and 0.797 mm, respectively, in the experiment and simulation. The predicted result is 0.80 mm for the truncated cone with the forming angle of 53.2°, which is equivalent to the result of sin(53.2°) = 0.8 mm calculated using the sine law.

It can be concluded from Figure 5 that (1) the experimental results are basically consistent with the theoretical prediction results and simulated results; (2) the thickness thinning occurs along the inclined wall; (3) the thickness of the inclined wall is basically the same indicating that the thickness is mainly determined by the forming angle, that is, the constant forming angle means constant thickness; (4) the thickness of the free zone on the edge side and the thickness of the non-deformation zone on the bottom surface are thinned less, and the deformation of the sheet is small; and (5) the thinnest thicknesses with forming angles of 76° and 53.2° are 0.948 mm and 0.765 mm in the experiment, respectively. The deviations from the predicted thicknesses are 0.022 mm and 0.036 mm, and the corresponding errors are 2.32% and 4.71%, respectively.

### 3.2. Case 2: A truncated Pyramid Part

A truncated pyramid as a NURBS surface of degree 1 in each of u and v with 5 × 3 control points can be constructed as

(9) $$P(x,y,z)=f(u,v)=\frac{\sum_{i=0}^{4}\sum_{j=0}^{2}N_{i,2}(u)N_{j,2}(v)\omega_{i,j}P_{i,j}}{\sum_{i=0}^{4}\sum_{j=0}^{2}N_{i,2}(u)N_{j,2}(v)\omega_{i,j}}\begin{array}{cc} & 0\leq u,v\leq 1 \end{array}$$

where the knot vectors are $U_{1\times 12}=\left[0,0,0.25,0.5,0.75,1,1\right]$ and $V_{1\times 6}=\left[0,0,0.5,1,1\right]$. The array of weights is $\left[\omega_{i,j}\right]=\left[\begin{array}{ccc} \begin{array}{ccc} 1 & 1 & 1 \end{array} & 1 & 1\\ \begin{array}{ccc} 1 & 1 & 1 \end{array} & 1 & \;1\;\\ \begin{array}{ccc} 1 & 1 & 1 \end{array} & 1 & 1 \end{array}\right]$.

**Figure 6:**
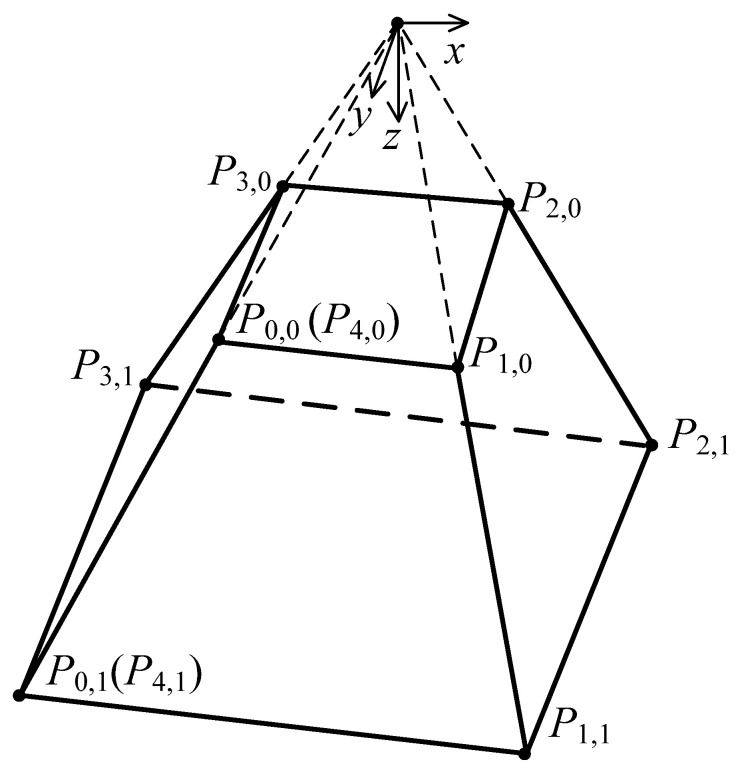
A truncated pyramid surface of degree 1 in each of its 2 parameters.

A truncated pyramid with forming angle of 53.2° shown in Figure 6 was designed by:

$$[P_{0,0},P_{0,1},P_{0,2}]=\left[\begin{array}{ccc} -10 & -30 & -30\\ 10 & 30 & 30\\ 7.5 & 22.5 & 22.5 \end{array}\right]$$

**Figure 7:**
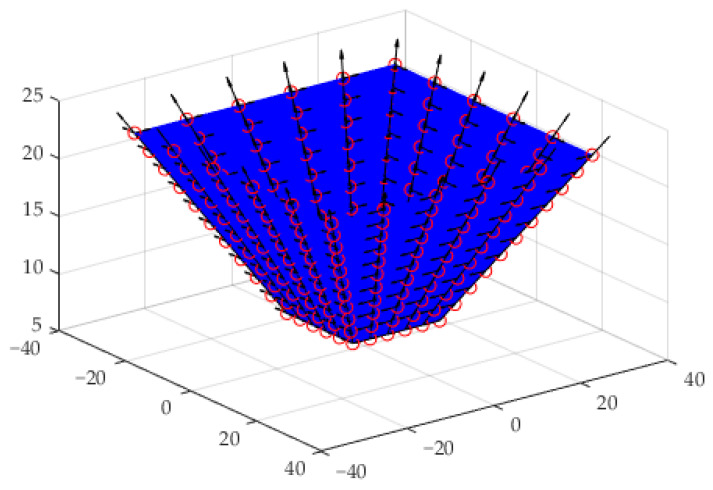
The first derivatives over the designed pyramid surface.

The first derivatives over the designed pyramid surface are shown in Figure 7.

**Figure 8:**
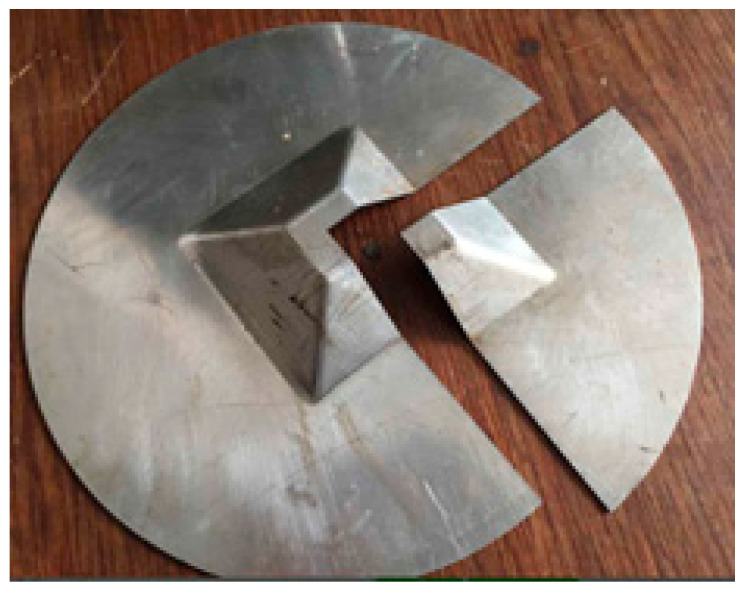
Formed truncated pyramid part.

The truncated pyramid, which was formed using aluminum sheet with thickness of 1 mm and axial step down 0.3 mm from Table 2, is presented in Figure 8.

**Figure 9:**
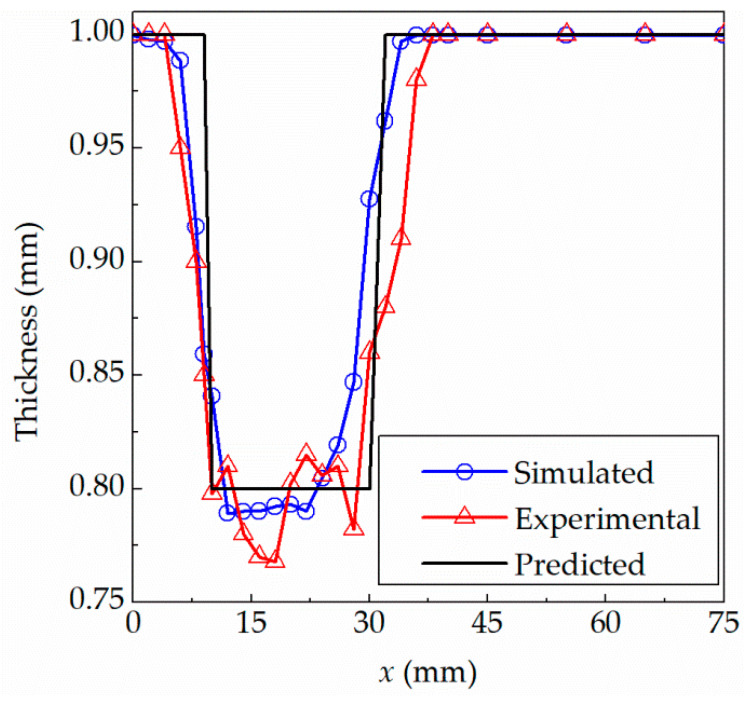
Comparison among experimental, simulated and theoretical predicted thickness distributions for the truncated pyramid.

Figure 9 shows the comparison among experimental, simulated and theoretical predicted thickness distributions for the truncated pyramid part. The average thicknesses of the inclined wall in the formed area within the range of 10–30 mm are 0.801 mm and 0.816 mm, respectively, in the experiment and simulation. The results predicted by this presented method and calculated by the traditional sine law are both 0.80 mm for the truncated pyramid part with the forming angle of 53.2°, which indicates that the result of the presented algorithm is completely consistent with the sine law for parts with a constant forming angle. In addition, compared with Figure 5, it can be seen that the smaller the forming angle, the greater the thickness reduction, and the thinner the final sheet, that is, the actual thickness is basically determined by the forming angle. The thicknesses of the inclined wall region are basically the same for the truncated cone and pyramid with the forming angle of 53.2°.

As shown in Figure 9, we can conclude that: (1) the experimental and simulated results are basically consistent with the theoretical prediction results; (2) the experimental thickness is basically the same as the original thickness in the unformed area and the experimental thickness is basically constant in the inclined wall area; and (3) the experimental thinnest thickness is 0.76 mm at 18 mm away from the center; the corresponding simulated and theoretical predicted thicknesses are 0.7923 mm and 0.8 mm, respectively. The deviations from the predicted thicknesses are 0.04 mm, and the corresponding errors are 5.26%.

### 3.3. Case 3: A Hemi-Ellipsoid Part

The construction of an ellipsoid as a NURBS surface with the degree of 2 in each of u and v is similar to that for the cone.

With 9 × 3 control points can be constructed as:

(10) $$P(x,y,z)=f(u,v)=\frac{\sum_{i=0}^{8}\sum_{j=0}^{2}N_{i,2}(u)N_{j,2}(v)\omega_{i,j}P_{i,j}}{\sum_{i=0}^{8}\sum_{j=0}^{2}N_{i,2}(u)N_{j,2}(v)\omega_{i,j}}\begin{array}{cc} & 0\leq u,v\leq 1 \end{array}$$

where the knot vectors are $U_{1\times 12}=\left[0,0,0,0.25,0.25,0.5,0.5,0.75,0.75,1,1,1\right]$ and $V_{1\times 6}=\left[0,0,0,1,1,1\right]$. The array of weights is $\left[\omega_{i,j}\right]=\left[\begin{array}{c} 1\\ \sqrt{2}/2\\ 1 \end{array}\begin{array}{c} \sqrt{2}/2\\ 0.5\\ \sqrt{2}/2 \end{array}\begin{array}{c} 1\\ \sqrt{2}/2\\ 1 \end{array}\begin{array}{c} \sqrt{2}/2\\ 0.5\\ \sqrt{2}/2 \end{array}\begin{array}{c} 1\\ \sqrt{2}/2\\ 1 \end{array}\begin{array}{c} \sqrt{2}/2\\ 0.5\\ \sqrt{2}/2 \end{array}\begin{array}{c} 1\\ \sqrt{2}/2\\ 1 \end{array}\begin{array}{c} \sqrt{2}/2\\ 0.5\\ \sqrt{2}/2 \end{array}\begin{array}{c} 1\\ \sqrt{2}/2\\ 1 \end{array}\right]$.

**Figure 10:**
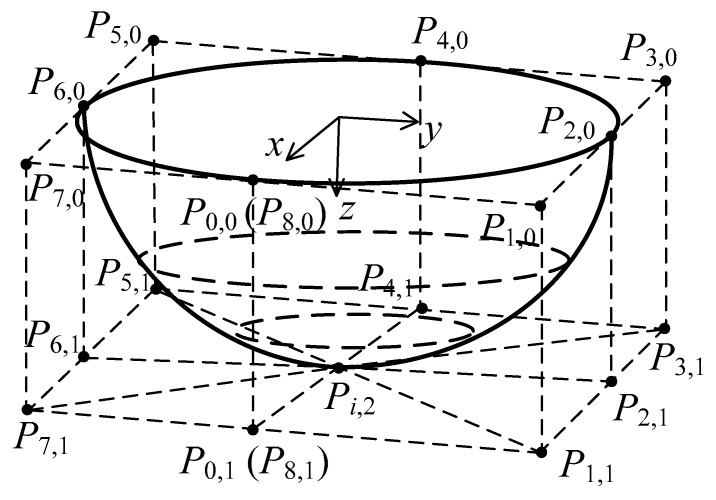
A hemi-ellipsoid surface of degree 2 in each of its 2 parameters.

A hemi-ellipsoid shown in Figure 10 was designed by:

$$[P_{0,0},P_{0,1},P_{0,2}]=\left[\begin{array}{ccc} 32 & 32 & 0\\ 0 & 0 & 0\\ 0 & 15 & 15 \end{array}\right]$$

It can also be expressed as:

(11) $$\frac{x^{2}}{32^{2}}+\frac{y^{2}}{24^{2}}+\frac{z^{2}}{15^{2}}=1,\;0\leq z\leq 15$$

**Figure 11:**
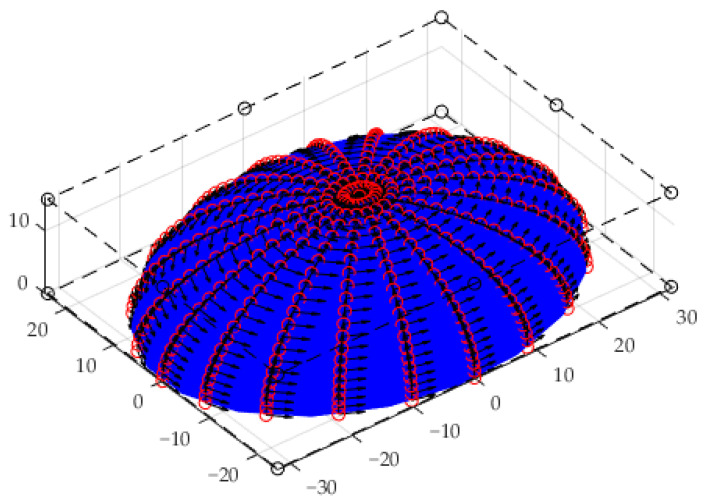
The first derivatives over the designed hemi-ellipsoid surface.

The first derivatives over the designed hemi-ellipsoid surface are shown in Figure 11.

**Figure 12:**
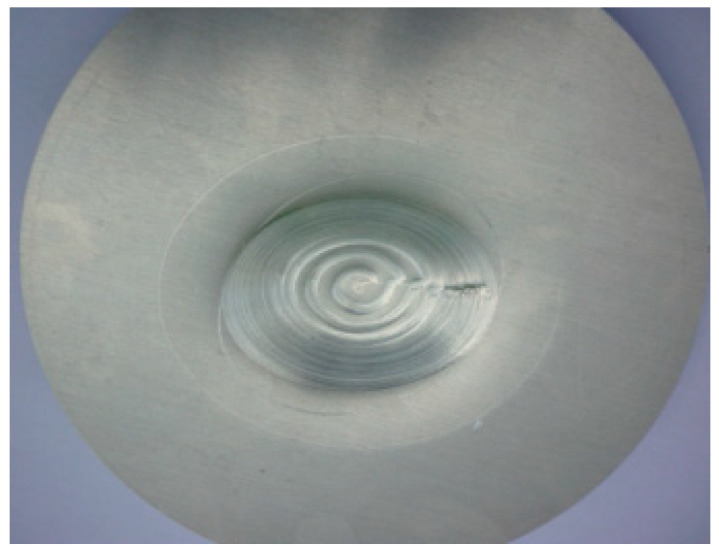
Formed hemi-ellipsoid part.

The hemi-ellipsoid, which was formed using Al1060 sheet with original thickness of 1 mm and the step-down 1 mm shown in Table 2, is presented in Figure 12.

**Figure 13:**
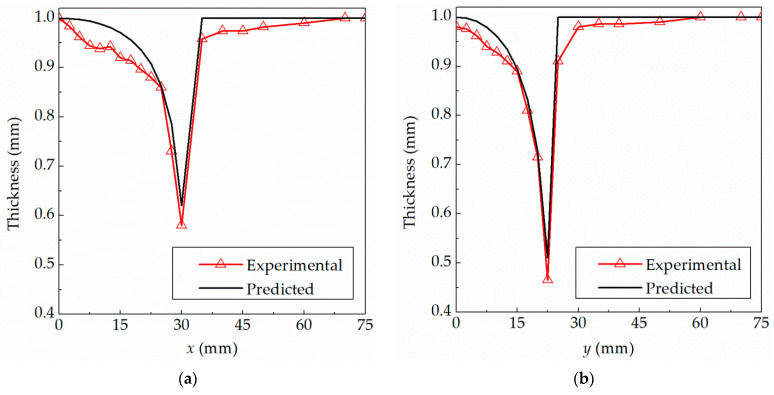
Comparison between experimental and theoretical predicted thickness distributions for hemi-ellipsoid: (**a**) the direction of major axis; (**b**) the direction of minor axis.

Figure 13 shows the thickness distributions of the experimental part and the theoretical result for the hemi-ellipsoid part. The curvature of an ellipsoidal surface is variable. Within the range of *x* = 0–30 mm along the major axis and *y* = 0–24 mm along the minor axis, the thickness of the sheet located in the formed region is varied. In the transition area where the length of the major axis exceeds 32 mm and the length of the minor axis exceeds 24 mm, the final thickness decreases slightly, because the deformation occurs in this area. In the small area near the center, the final thickness is basically close to the original thickness, indicating that the sheet in the area is mainly translated downward during the forming process, and is not directly processed by the forming tool. The minimum thicknesses along the major axis and minor axis occur near the outer edge of the ellipsoid. It should be noted that the final thickness of the formed hemi-ellipsoid part at any position can be predicted by the presented algorithm in this paper. However, it is very difficult to calculate the thickness of the parts with the varying curvature by using the traditional sine law.

As shown in Figure 13, we can conclude that: (1) the experimental and theoretical thickness profiles are basically consistent; (2) the final thickness differs significantly with the alteration of the forming angle; and (3) the experimental thinnest thicknesses are 0.580 mm and 0.465 mm, respectively, at 30 mm and 22.5 mm away from the center along the direction of major and minor axes; the corresponding theoretical predicted thicknesses are 0.6208 mm and 0.5106 mm, respectively. The deviations between the experimental and theoretical thickness are 0.0408 mm and 0.0456 mm, respectively, and the corresponding errors are 7.03% and 9.81%, respectively.

In order to have a further understanding of the presented model, the maximum deviation and root mean square error (RMSE) between the predicted, experimental and simulated thicknesses shown in Figure 5, Figure 9 and Figure 13 are summarized, calculated and listed in Table 3.

The following points can be seen from Table 3. (1) For all formed parts, the maximum deviation is only 0.0456 mm, and the corresponding percentage is 9.81%, which indicates that the deviation of prediction is basically small. It can be concluded that the thickness of theoretical prediction matches well with that of experiment and simulation within the acceptable deviation. (2) With the forming part deeper and the surface feature more complex, the prediction error will be larger. (3) The maximum thinning often occurs in the transition region between the formed area and the unformed area. (4) The maximum RMSE between the experimental and simulated thicknesses which occurs for the formed truncated pyramid is 0.0336 mm. The maximum RMSE between the predictions and experimental data, finite element results are 0.0499 mm and 0.0539 mm, respectively. The results show that the simulated thickness is closer to experimental thickness than that of the prediction, and the proposed model in this paper can effectively predict the thickness of parts in incremental sheet metal forming.

## 4. Conclusions

In summary, a general algorithm to predict and calculate the final thickness for arbitrary shape parts in incremental sheet metal forming based on a NURBS surface is presented. A detailed analysis is accomplished for the truncated cones, pyramid and ellipsoid parts. The experimental tests based on the customized deformation machine and finite element simulations using ANSYS/LS-DYNA are conducted. The comparison of the thickness of three kinds of formed parts shows that the maximum deviation and maximum RMSE are 0.0456 mm (9.81%) and 0.0539 mm, respectively. It is also found that the maximum deviation in the case of a constant forming angle is smaller than that of a variable forming angle. With the increase in depth and feature complexity, the deviation increases. The prediction results agree well with the experimental measurements and finite element results, which verifies that the proposed mathematical modeling approach can well predict the thickness distributions of the parts manufactured by using incremental sheet metal forming. Compared with the traditional sine law, this presented method can predict and calculate the thickness profile of arbitrary complex shape parts with variable curvature surfaces such as spherical and ellipsoidal parts in the design stage. This work provides a promising method to prevent thickness thinning during sheet metal forming.

## Figures and Tables

**Table 1:** Material parameters of aluminum sheet and forming tool.

Material Parameters	Aluminum Sheet	Forming Tool
Density	2700 kg m^−3^	7850 kg m^−3^
Elastic modulus	69 GPa	210 GPa
Poisson’s ratio	0.33	0.3

**Table 2:** Some forming parameters.

	Radial Feed Rate	Axial Step Down	Rotational Speed
Truncated cone (76°)	2.0 mm	0.5 mm	4.0 r/min
Truncated cone (53.2°)	1.0 mm	0.75 mm	4.0 r/min
Truncated pyramid	0.4 mm	0.3 mm	2.0 r/min
Hemi-ellipsoid	0.3–4.4 mm	1.0 mm	0.5 r/min

**Table 3:** The maximum deviation and RMSE between the predicted, experimental and simulated thicknesses.

Formed Parts	Depth	Maximum Deviation of Thickness	Percentage	RMSE between Prediction and Experiment	RMSE between Prediction and Simulation	RMSE between Experiment and Simulation
Truncated cone (76°)	5 mm	0.0220 mm	2.32%	0.0197 mm	0.0148 mm	0.0061 mm
Truncated cone (53.2°)	15 mm	0.0360 mm	4.71%	0.0471 mm	0.0539 mm	0.0157 mm
Truncated pyramid	15 mm	0.0400 mm	5.26%	0.0499 mm	0.0437 mm	0.0336 mm
Hemi-ellipsoid (major axis)	15 mm	0.0408 mm	7.03%	0.0339 mm	-	-
Hemi-ellipsoid (minor axis)	15 mm	0.0456 mm	9.81%	0.0305 mm	-	-

## Data Availability

All data is presented within the manuscript.
